# Prognostic Value of NME1 (NM23-H1) in Patients with Digestive System Neoplasms: A Systematic Review and Meta-Analysis

**DOI:** 10.1371/journal.pone.0160547

**Published:** 2016-08-12

**Authors:** Wei Han, Chun-tao Shi, Fei-yun Cao, Fang Cao, Min-bin Chen, Rong-zhu Lu, Hua-bing Wang, Min Yu, Da-wei He, Qing-hua Wang, Jie-feng Wang, Xuan-xuan Xu, Hou-zhong Ding

**Affiliations:** 1 Department of General Surgery, Kunshan First People's Hospital Affiliated to Jiangsu University, Kunshan Jiangsu, 215300, P. R. China; 2 Department of General Surgery, Xishan People’s Hospital, Wuxi Jiangsu, 215300, P. R. China; 3 School of Medicine, Jiangsu University, Zhenjiang Jiangsu, 212001, P. R. China; 4 Department of Radiotherapy and Oncology, Kunshan First People's Hospital Affiliated to Jiangsu University, Kunshan Jiangsu, 215300, P. R. China; 5 Laboratory Department, Kunshan First People's Hospital Affiliated to Jiangsu University, Kunshan Jiangsu, 215300, P. R. China; 6 Digestive System Department, Kunshan First People's Hospital Affiliated to Jiangsu University, Kunshan Jiangsu, 215300, P. R. China; 7 Department of General Surgery, Qiandeng Hospital, Kunshan Jiangsu, 215300, P. R. China; National Taiwan University College of Medicine, TAIWAN

## Abstract

**Objective:**

There is a heated debate on whether the prognostic value of NME1 is favorable or unfavorable. Thus, we carried out a meta-analysis to evaluate the relationship between NME1 expression and the prognosis of patients with digestive system neoplasms.

**Methods:**

We searched PubMed, EMBASE and Web of Science for relevant articles. The pooled odd ratios (ORs) and corresponding 95%CI were calculated to evaluate the prognostic value of NME1 expression in patients with digestive system neoplasms, and the association between NME1 expression and clinicopathological factors. We also performed subgroup analyses to find out the source of heterogeneity.

**Results:**

2904 patients were pooled from 28 available studies in total. Neither the incorporative OR combined by 17 studies with overall survival (OR = 0.65, 95%CI:0.41–1.03, P = 0.07) nor the pooled OR with disease-free survival (OR = 0.75, 95%CI:0.17–3.36, P = 0.71) in statistics showed any significance. Although we couldn’t find any significance in TNM stage (OR = 0.78, 95%CI:0.44–1.36, P = 0.38), elevated NME1 expression was related to well tumor differentiation (OR = 0.59, 95%CI:0.47–0.73, P<0.00001), negative N status (OR = 0.54, 95%CI:0.36–0.82, P = 0.003) and Dukes’ stage (OR = 0.43, 95%CI:0.24–0.77, P = 0.004). And in the subgroup analyses, we only find the “years” which might be the source of heterogeneity of overall survival in gastric cancer.

**Conclusions:**

The results showed that statistically significant association was found between NME1 expression and the tumor differentiation, N status and Dukes’ stage of patients with digestive system cancers, while no significance was found in overall survival, disease-free survival and TNM stage. More and further researches should be conducted to reveal the prognostic value of NME1.

## Introduction

Digestive system neoplasms, including colorectal cancer, gastric cancer, esophageal cancer, pancreatic cancer, hepatocellular carcinoma, and gallbladder carcinoma, with the high morbidity and mortality, have become one of the most terrible threat for human beings[1]. Despite plenty of biomarkers involved in digestive system neoplasms have been identified, the prognosis remains to be dismal mainly due to local recurrence, lymph node invasion and distant metastasis[2]. Besides, patients at the same status, for instance tumor differentiation, lymph node metastases and TNM stage, may have diverse clinical outcomes[3]. Thus, it is urgent to develop new reliable prognostic markers to predict the prognosis and supply better and more suitable therapy for patients with digestive system neoplasms.

NME1 (also known as NM23-H1 and NDPK-A), the first metastasis suppressor protein of the ten members of NM23 family[4] (NM23 stands for non-metastatic clone 23), has been found associated with the development and progression of various neoplasms[5,6,7]. After transplanting eight ovarian cancer cell lines subcutaneously into the flank of nude mice, the expression of NME1 mRNA and protein in human ovarian cancer cells was inversely related to metastatic behavior in experimental animals (r = 0.96, P = 0.0001)[8]. Transfection into melanoma cell lines also inhibited invasion, motility, colonization, differentiation and liver metastasis[9]. McCorkle investigated NME1-regulated gene expression in WM1158 and WRO82 cells and found that a number of genes regulated by NME1 in melanoma and thyroid carcinoma cell lines would become potential predictors of survival in breast cancer[10]. When comparing the primary two members of NM23 family, Tokunaga found that the expression of NME1, but not NME2, was inversely associated with lymph-node metastasis (p < 0.01)[11]. In digestive system tumors, NME1 also plays an critical role in many respects. Boissan[12] discovered that, at early stages of the invasive program, NME1 could control the cell-cell adhesion and cell migration. After silencing NME1 expression in human hepatoma and colon carcinoma cells, cellular scattering, motility, and extracellular matrix invasion were all promoted[12]. Moreover, NME1 may act as a molecular switch between the free-floating and adherent states of gastric cancer cells[13].

The expression of NME1 has been reported to be a promising prognostic indicator. Most studies reported that over-expression of NME1 was associated with a better overall survival of various cancers, like liver, colorectal, breast, lung, and esophageal cancers. However, some studies showed that NME1 was not a metastasis suppressor gene and not correlated with metastasis[14,15]. In addition, none of these reports have been confirmed by systematic reviews with meta-analysis. Therefore, to clarify this question and explore its prognostic value, we performed this systematic review of the literature with meta-analysis.

## Materials and Methods

### Database search strategy

We performed systematic literature search of Pubmed, EMBASE and Web of Science from their incipiency to October, 2015. The retrieval strategy was used as follow: (NME1 or (non-metastasis 23-H1) or (nucleoside diphosphate kinase A) or NDPK-A or NME1) and (digestive system or esophagus or oesophagus or gastric or stomach or colorectal or colonic or rectal or gastrointestinal or gastroenteric or pancreatic or hepatocellular or hepatic or ampulla or ampullary or gallbladder) and (neoplasms or cancer or carcinoma or tumor or tumour or adenocarcinoma or malignant) and (prognosis or prognostic or predict or survival or outcome or prognos* or (clinical variables) or clinicopatholog* or (clinical pathology) or (clinic pathology)). Reference lists of articles and reviews were hand-searched for additional studies. Manuscripts were also manually scanned to obtain potential articles most relevant to this review. Only studies published in peer reviewed journals were included. The language of all studies was limited to English. All the initially identified articles were scrutinized independently by two reviewers (Wei Han and Chun-tao Shi). For more details and for information, please see our protocol with the registration number: CRD42015029269[16].

### Inclusion criteria

To be eligible for inclusion, the following criteria had to be fulfilled: (a) clinical studies researched patients with digestive system cancers; (b) NME1 expression in cytoplasm of tissue specimens of patients with digestive system cancers, who received neither chemotherapy nor radiation therapy before surgery, was measured with immunohistochemistry (IHC); (c) studies reported the association between NME1 expression and survival outcome or clinicopathological information; (d) only the most recent or the most complete report would be enrolled, if the study population was duplicated or overlapping. Disagreement was resolved by discussion between the two reviewers or consultation with a third reviewer (Min-bin Chen).

### Exclusion criteria

Exclusion criteria were: (a) literature published as letters, editorials, abstracts, reviews, case reports and expert opinions; (b) experiment in vitro or in vivo but not based on patients; (c) articles without the ORs with 95% CI about clinicopathological information, or the Kaplan-Meier survival curves; (d) repeated and similar studies.

### Data extraction

The following information from each article was extracted: (a) general information, including first author, publication year, country (area) of origin, age and gender of the study patients, sample size and the follow-up duration; (b) clinicopathological characteristics, including TNM staging, Dukes’ stage, differential grade and lymph node metastasis/N status; (c) method to determine NME1 expression and number of patients stratified by NME1 expression; (d) clinical outcomes, including OS or DFS and its correlative ORs with 95%CI, which were all estimated from Kaplan-Meier curves.

### Quality assessment

Two independent reviewers (Wei Han and Chun-tao Shi) assessed the quality of each study with the Newcastle-Ottawa Quality Assessment Scale (NOS)[17] which was mainly used in retrospective studies. A study with NOS ≥ 6 was regarded as a high-quality study[18]. Disparity was resolved by discussion or consultation.

### Data synthesis and analysis

Overall survival (OS) and disease-free survival (DFS) associated with NME1 expression in patients with digestive system cancers, were the primary outcomes. The secondary outcome was the relationship between the clinicopathological factors and the expression of NME1. OR with its 95% CI was used to be the effect measure of interest. Estimates of ORs were weighted and pooled using the Mantel-Haenszel method. A combined OR>1, with its 95% CI did not overlap 1, indicated a worse survival for the group with NME1 expression. The heterogeneity among studies was measured using the Q and I^2^ test. A random or Fixed model was used according the heterogeneity analysis. A random effect model was applied if I^2^≧50%; the fixed effect model was selected if I^2^<50%. There was substantial heterogeneity in studies if an I^2^>50%, and we would carry out subgroup analysis to fine the source of heterogeneity. A P < 0.05 indicates a significant factor contributing to the observed heterogeneity. The latent publication bias was assessed by a funnel plot and Egger’s linear regression test, and a value <0.05 indicated an inevitable significant publication bias[19]. All statistical tests were two-tailed and P<0.05 was considered statistically significant. All the analyses were conducted by Review Manager software version 5.3 (The Cochrane Collaboration) and STATA statistical software package version 12.0 (Stata Corporation, College Station, TX).

## Results

### Literature search

**Fig 1:**
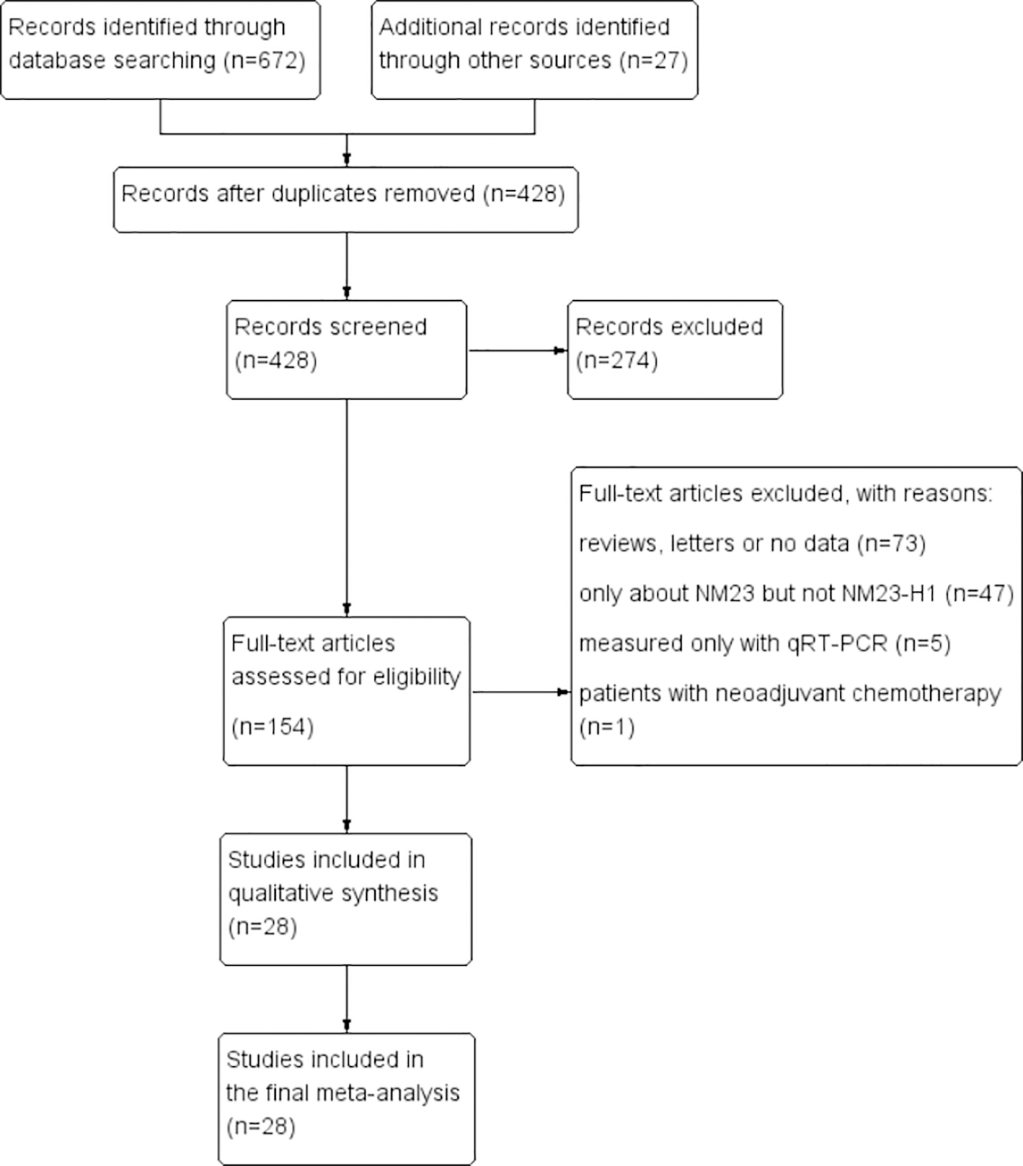
Flow chart for the selection of records to include.

A total of 672 articles were retrieved in the initial search of databases. In addition, 27 records were yielded by manual searching. After removing 271 duplicates, we read the titles and abstracts of the 428 studies left. 274 citations were excluded from analysis based upon abstracts or titles, leaving 154 studies for further full-text review. After meticulously reading, 124 studies were excluded: 73 studies, including reviews or letters, were excluded for no or insufficient survival data; 47 left were excluded in that they were only about NM23, but not NME1; four studies were measured only with qRT-PCR but not IHC; and the left one reported the patients with neoadjuvant chemotherapy[20]. As a result, 28 eligible studies[21–48] with 2904 patients in total, were enrolled in this meta analysis (Fig 1).

### Study characteristics

The basic characteristics of the 28 studies[21–48], published ranging from 1993 to 2012, are summarized in Table 1. Briefly, study sample sizes ranged from 25 to 413; 21 studies were conducted in Asian populations, while the remaining used Caucasian populations; colorectal cancer (CRC), gastric cancer (GC), esophagus cancer (EC), hepatocellular carcinoma (HCC), pancreatic cancer (PC) and gallbladder carcinoma (GBC) were studied in 13, 8, 4, 3, 2 and 1 articles, respectively; all studies measured the expression of NME1 in cytoplasm of tissue specimens with IHC, and all patients didn’t receive any preoperative chemotherapy or radiation therapy, as we had written before; all of the primary antibodies were anti-NME1 antibodies, including polyclonal and monoclonal antibodies. Except two articles[24,30], all of the other reported their cut-off of NME1 expression, most of which identified more than about 50% staining cancer cells as high expression. One study[43] reported that if more than 20% of the cancer cells were more strongly stained than stromal cells, they were considered positive, and the another one[45] regarded similar to or more intense than that of the adjacent nontumorous tissue as high expression. Although the cut-offs of these two studies were different from that of other studies, the effect, to some extent, is similar to more than 50%. However, the cut-offs of another three studies[33,34,47] might be too low as compared with others. We also found that Iizuka was the first author of two enrolled studies[42,43] with different population in the same period. So, we marked them as Iizuka1[42] and Iizuka2[43].

**Table 1:** Characteristics of included studies

First author	Year	NOS	Study region	N. of P.	Type	cut-off of NME1 high expression	Primary antibody	Follow-up time Mean (range)	Survival analysis
Lee^[21]^	2001	7	Taiwan	146	CRC	More than 50% or “++”	Monoclonal anti-NME1 antibody (Santa Cruz Biotechnology, Inc., Santa Cruz, CA)	54 months (3-91) months	OS
Tabuchi^[22]^	1999	6	Japan	52	CRC	Positive reactivity for strong staining	mouse monoclonal antihuman NME1 antibody (H1-229, 2µg/ml, Seikagaku, Tokyo, Japan)	> 5 years	OS
Lindmark^[23]^	1996	7	Sweden	202	CRC	strong and moderate homogeneous intensity	Mouse monoclonal anti-NME1 antibody, cloned NM301, from Becton and Dickinson(San Jose, CA, USA)	> 90 months	OS
Abad^[24]^	1996	5	Austria	62	CRC	NR	monoclonal antibody NCL-nm23-2	6 ~ 10 years	OS、DFS
Cheah^[25]^	1998	7	Singapore	141	CRC	moderate and strong staining	monoclonal antibody (NM23 Ab-1, clone NM301 from Oncogene Science)	> 5 years	OS、DFS
Chen^[26]^	2007	6	China	103	CRC	moderate and marked staining	Mouse anti-human monoclonal antibodies to NME1 (1:50dilution;ShanghaiChang-DoBiotechnology Co. Ltd)	NR	NR
Dursun^[27]^	2001	8	Turkey	185	CRC	More than 60%	prediluted primary polyclonal antibody (NDPKinase/nm23Ab-1,NeoMarkers,US)	36 months(2-95) months	OS、DFS
Kapitanovic^[28]^	2004	7	Croatia	73	CRC	On the basis of the relative visual intensity of chromogenic label	mouse monoclonal antibody to human NME1 (NM301 monoclonal antibody; Molecular Oncology Inc, Gaithersburg, Maryland, USA)	about 300 weeks	OS
Martinez^[29]^	1995	6	France	35	CRC	signal more intense than in matched normal tissue	anti-NDP kinase A monoclonal antibody (HA-37.6)raised by Hybridolab, Pasteur Institute, Paris	NR	NR
Su^[30]^	2004	5	China	30	CRC	NR	anti-NME1 antibody	NR	NR
Tannapfel^[31]^	1995	6	Germany	100	CRC	More than 60%	A 1:200 dilution of nm23Ab-1,Clone NM301,obtained from Oncogene Science Cambridge, MA	NR	NR
Yamaguchi^[32]^	1993	6	Japan	36	CRC	strongly stained	the primary antibody to NME1 (mAb HI -229)	NR	NR
Kim^[33]^	1995	6	Korea	101	GC	a few cells or more were positive	NDPK-A/nm23, Novocastra, 1:100 dilution, Newcastle upon Tyne, UK	NR	NR
Muller^[34]^	1998	8	Germany	413	GC	More than 1%	Polyclonal antibody (Boehringer Mannheim, Mannheim, Germany) that was raised against the NME1/NDP kinase A	2.3 years(2months- 9.1years)	OS
Oue^[35]^	2007	7	Japan	124	GC	more than 50%	rabbit polyclonal antiNME1 (1:20,Santa Cruz Biotechnology,Santa Cruz,CA, USA)	> 1500 days	OS
Su^[36]^	2001	8	China	59	GC	More than 50% or “+++”	Mouse monoclonal antibody against NME1 (NM301)	75months (60-96 months)	OS
Terada^[37]^	2002	8	Japan	103	GC	all of the epithelial cells in the lesion showed cytoplasmic staining	anti-nm23 monoclonal antibody (Diagnostic BioSystems, Flemont Blvd, CA), which specifically recognizes NME1	> 5 years	OS
Wang^[38]^	1998	7	Taiwan	37	GC	More than 75%	polyclonal antibodies(NME1 and SC343, Santa Cruz Biotechnology,Santa Cruz, CA)	About 2 years	OS
Yoo^[39]^	1999	7	Korea	261	GC	more than 30% stained with moderate or strong intensity	mouse monoclonal antibody raised against NDP-kinase A purified from humanerythrocytes(NCL-nm23, Novocastra Lab.,Newcastle-upon-Tyne,UK)	63months( 6-124months)	OS
Tomita^[40]^	2001	8	Japan	45	EC	More than 50%	The specific monoclonal antibody against NME1 gene product (Novocastra Laboratories, Newcastle, UK)	> 6 years	OS
Wang^[41]^	2004	7	Taiwan	145	EC	More than 20%	Monoclonal antibody specific to NME1 was manufactured at Santacruz (CA,USA), and a dilution of 1:50 was applied	> 65 months	OS
Iizuka 1^[42]^	1999	8	Japan	50	EC	staining was more intense than stromal cells	antihuman NME1 monoclonal antibody (H1-229, Seikagaku Corp., Tokyo, Japan)	63 months(21±105)months	OS
Iizuka 2^[43]^	1999	8	Japan	32	EC	> 20% of the cancer cells were more strongly stained than stromal cells	anti-human NME1 monoclonal antibody(H1-229,Seikagaku,Tokyo, Japan)(Tokunaga et al, 1993; Iizuka et al, 1995)	65months (21-105)months	OS、DFS
Liu^[44]^	2005	6	China	33	HCC	More than 30%	mouse NME1 monoclonal antibody	6-16 months	NR
Yamaguchi^[45]^	1994	6	Japan	25	HCC	similar to or more intense than that of the adjacent nontumorous tissue	specific monoclonal antibodies directed against NME1 protein(monoclonal antibody [MoAb] H1-229)	< 4 years	NR
Ohshio^[46]^	1997	6	Japan	73	PC	More than 34% or “++/+++”	Monoclonal anti-nm23 antibody (clone 37.6, IgG2a) immunizing with NDP kinase A (NME1)	< 800 days	NR
Takadate^[47]^	2012	7	Japan	73	PC	More than 10%	Mouse monoclonal nm23/nucleoside diphosphate kinase-A (Nm23/NDPK-A)antibody, clone37.6 (Abcam, MA, USA) ata 1:100 dilution	about 60 months	OS、DFS
Yang^[48]^	2008	6	China	165	GCCRCHCCGBC	Excel function to compute the value of positive unit (PU)	anti-NME1 antibody	NR	NR

### Quality assessment

The study quality scores based on the NOS, ranged from 5 to 8, with a mean of 6.75. Only two of these 28 studies gained a NOS = 5 (< 6), suggesting that only these two studies had low quality, and the other had high levels of methodological quality in this meta-analysis (Table 2).

**Table 2:** Quality assessment with Newcastle-Ottawa Scale.

First author	Year	NOS	Selection	Comparability	Outcome
Lee	2001	7	★★★*	★★	★★*
Tabuchi	1999	6	★★★	★*	★★
Lindmark	1996	7	★★★	★★	★★
Abad	1996	5	★★*	★*	★★
Cheah	1998	7	★★★*	★★	★★*
Chen	2007	6	★★★	★	★★
Dursun	2001	8	★★★*	★★*	★★★
Kapitanovic	2004	7	★★★	★★	★★
Martinez	1995	6	★★★	★	★★*
Su	2004	5	★★★*	★*	★*
Tannapfel	1995	6	★★★	★	★★
Yamaguchi	1993	6	★★	★★	★★*
Kim	1995	6	★★★*	★	★★
Muller	1998	8	★★★	★★*	★★★
Oue	2007	7	★★★*	★★	★★*
Su	2001	8	★★★*	★★*	★★★*
Terada	2002	8	★★★	★★	★★★
Wang	1998	7	★★★	★★	★★*
Yoo	1999	7	★★*	★★	★★★*
Tomita	2001	8	★★★	★★*	★★★*
Wang	2004	7	★★★	★*	★★★
Iizuka 1	1999	8	★★★	★★	★★★
Iizuka 2	1999	8	★★★*	★★	★★★
Liu	2005	6	★★★	★	★★*
Yamaguchi	1994	6	★★	★*	★★★
Ohshio	1997	6	★★★	★	★★
Takadate	2012	7	★★*	★★	★★★*
Yang	2008	6	★★★*	★*	★★

* The score was produced by the joint discussion; others were assessed by Wei Han and Chun-tao Shi, individually.

### Relationship of NME1 expression with survival

**Fig 2:**
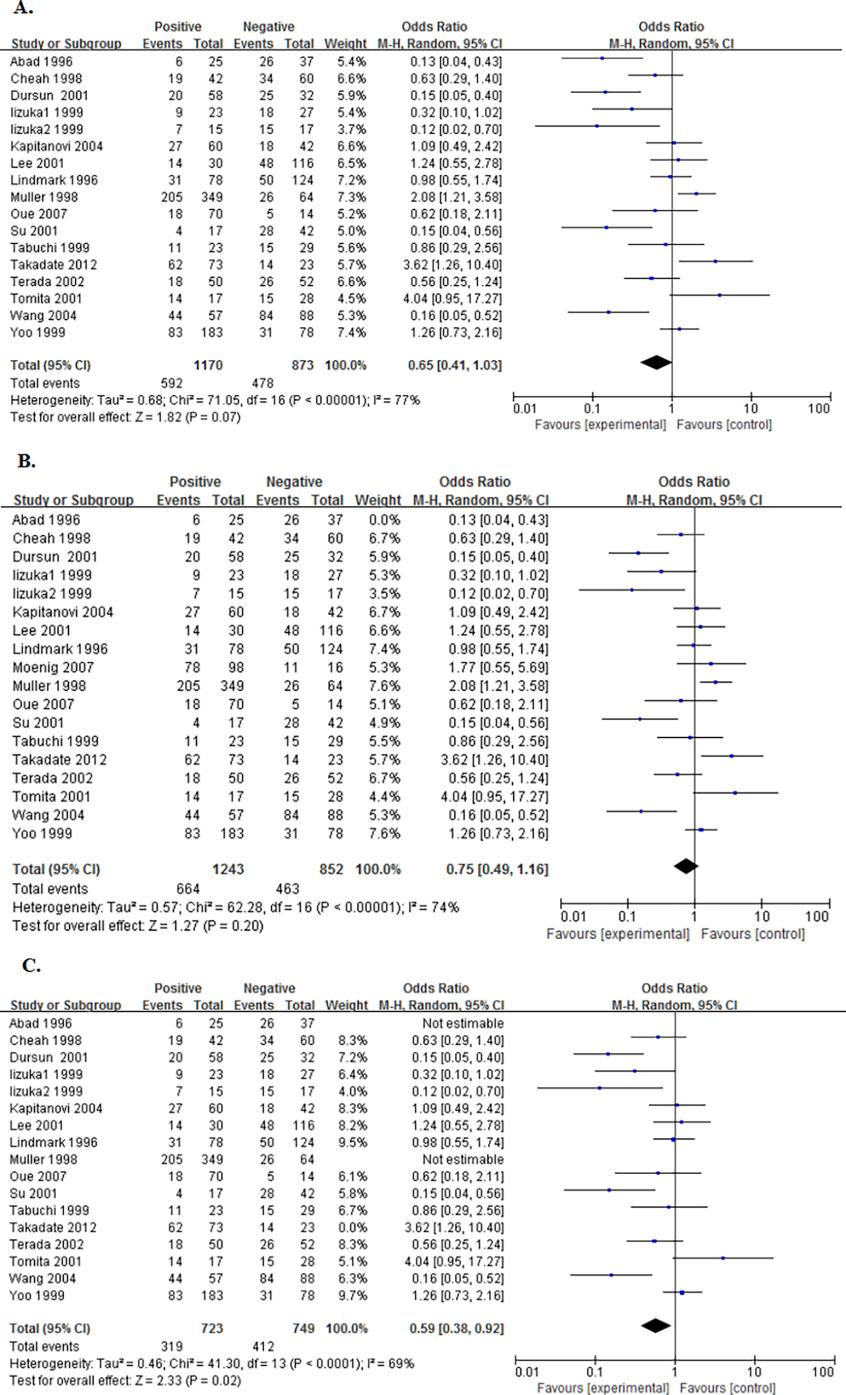
Forest plot of odds ratio (OR) for the association between NME1 overexpression and overall survival (OS) in patients with digestive system cancers with random effects model. A. The ORs for OS; B. The ORs for OS without “Abad 1996”; C. The ORs for OS without low cut-offs.

17 studies reported the data concerning the association between NME1 expression and overall survival (OS) of the patients. The pooled OR being 0.65 (95%CI:0.41–1.03, P = 0.07. Fig 2A) showed that there was no significance between the expression of NME1 and OS. Likewise, when we deleted this study[24], Abad 1996, which had a NOS<6, the new pooled OR being 0.75 (95%CI:0.49–1.16, P = 0.20. Fig 2B) also showed no significance in statistics. Then, we removed the another two studies[34,47], whose cut-offs were too low as compared with others. Though with heterogeneity (I² = 69%, P value of Q test for heterogeneity test (Ph) < 0.0001), this new pooled OR being 0.59 (95%CI:0.38–0.92, P = 0.02. Fig 2C) suggested that elevated NME1 expression predicted better OS.

**Fig 3:**
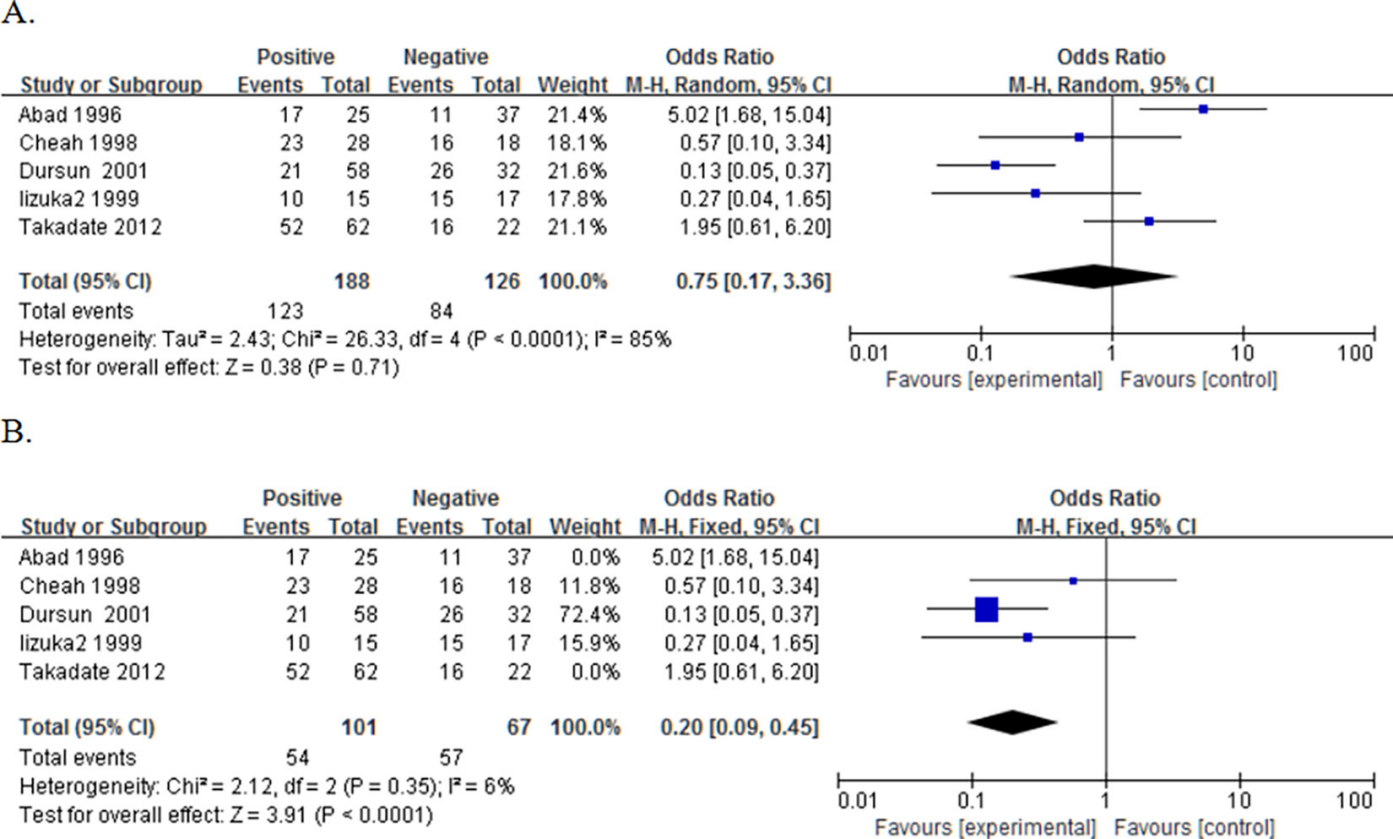
Forest plot of odds ratio (OR) for the association between NME1 overexpression and disease-free survival (DFS) in patients with digestive system cancers. A. The ORs for DFS; B. The new ORs for DFS.

Five cohorts presented the data of NME1 expression and disease-free survival (DFS) of the enrolled patients. Also, there was no significance with a pooling OR being 0.75 (95%CI:0.17–3.36, P = 0.71. Fig 3A). After deleting the two studies[24,47], one with a low NOS score and not reporting the cut-off, and the other with a low cut-off, a new pooled OR being 0.20 (95%CI:0.09–0.45, P<0.0001. Fig 3B), without heterogeneity (I^2^ = 6%, Ph = 0.35), showed that the overexpression of NME1 predicted better DFS.

### Relationship of NME1 expression with survival by tumor type

**Fig 4:**
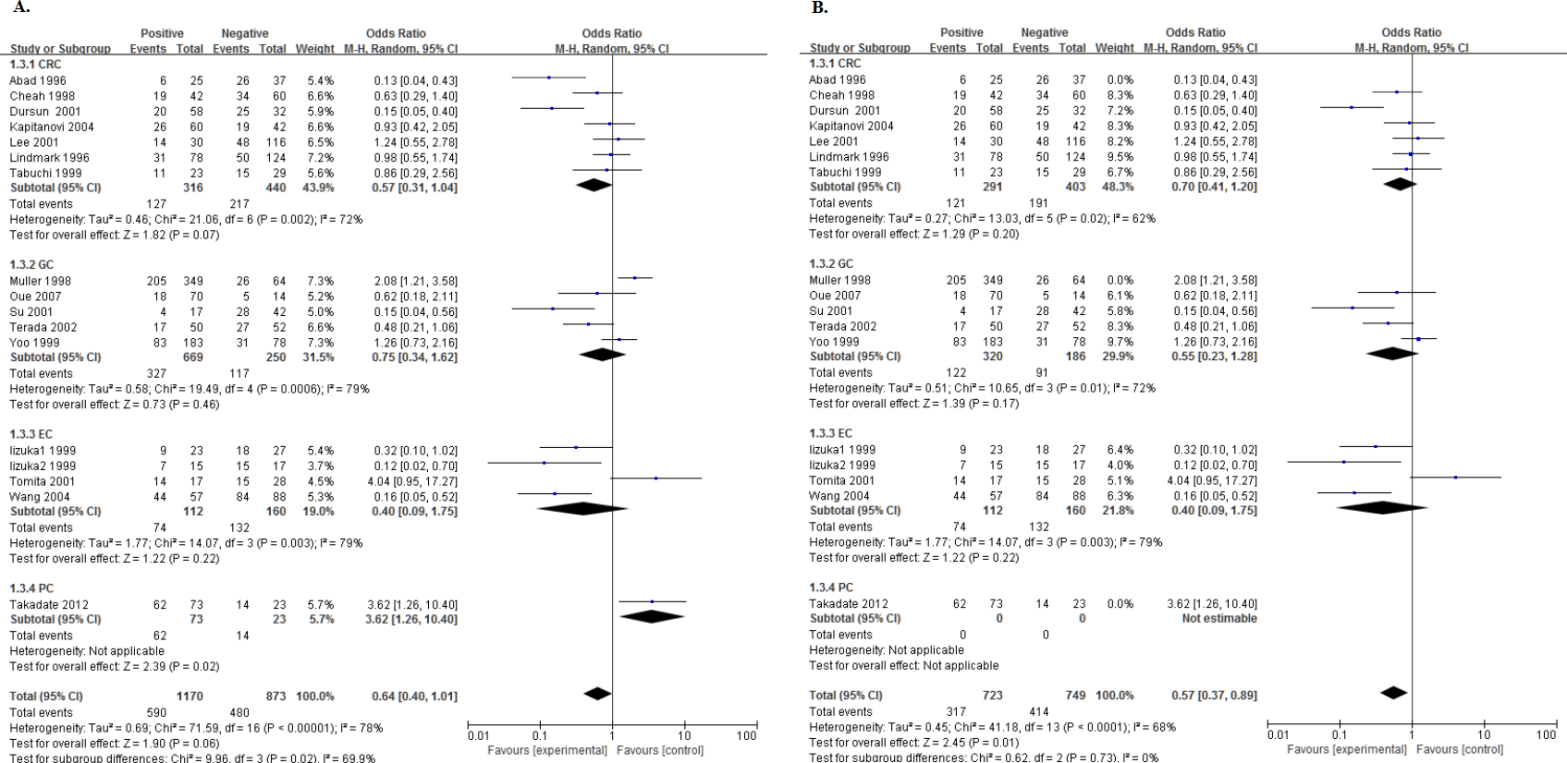
Forest plot of odds ratio (OR) for the association between NME1 overexpression and overall survival (OS) in patients with different tumor types with random effects model. A. The ORs for OS; B. The new ORs for OS.

There were 7, 5, 4 and 1 studies reporting the data of NME1 expression and overall survival (OS) of the patients with colorectal cancer, gastric cancer, esophagus cancer and pancreatic cancer, respectively. However, except that the only one study[47] which reported the data in PC, couldn’t be combined, all of the pooling ORs in other three tumor types had no significance in statistics (Fig 4A). Then, we deleted the three studies[24,34,47] as before. Though with significance in total (OR = 0.57, 95%CI:0.37–0.89, P = 0.01), none of these three types had a P<0.05 (Fig 4B).

**Fig 5:**
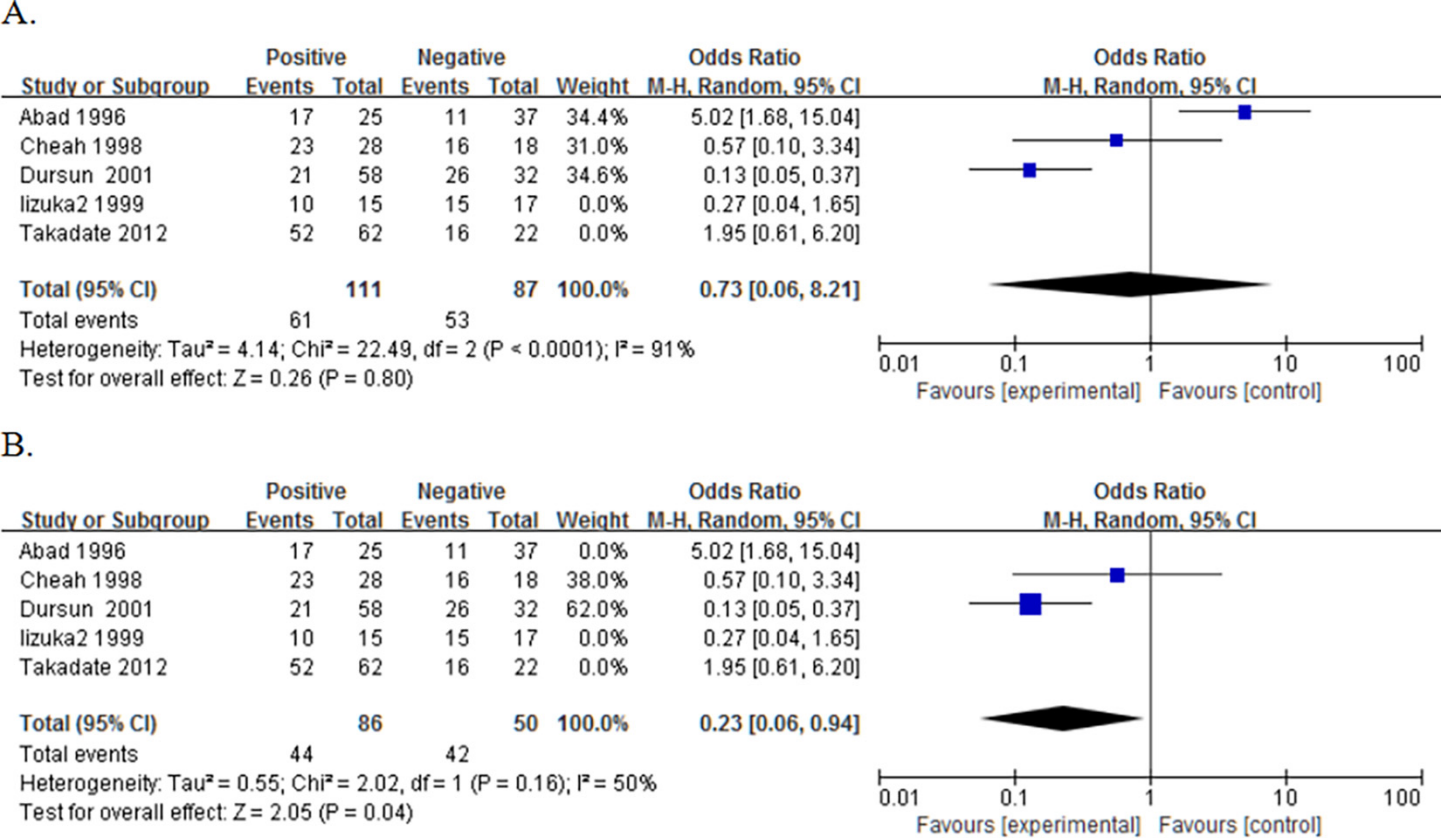
Forest plot of odds ratio (OR) for the association between NME1 overexpression and disease-free survival (DFS) in patients with different tumor types with random effects model. A. The ORs for DFS; B. The new ORs for DFS.

As for DFS, there were only three studies could be combined[24,25,27], all of which reported the data in CRC. However, we also found no significance in this type (OR = 0.73, 95%CI:0.06–8.21, P = 0.80. Fig 5A). Then, we deleted the one[24] with a low NOS score. Because of the I^2^ = 50%, we used the random effects model and gained a pooled OR being 0.23 (95%CI:0.06–0.94, P = 0.04. Fig 5B).

### Relationship of NME1 expression with clinical pathological factors

**Fig 6:**
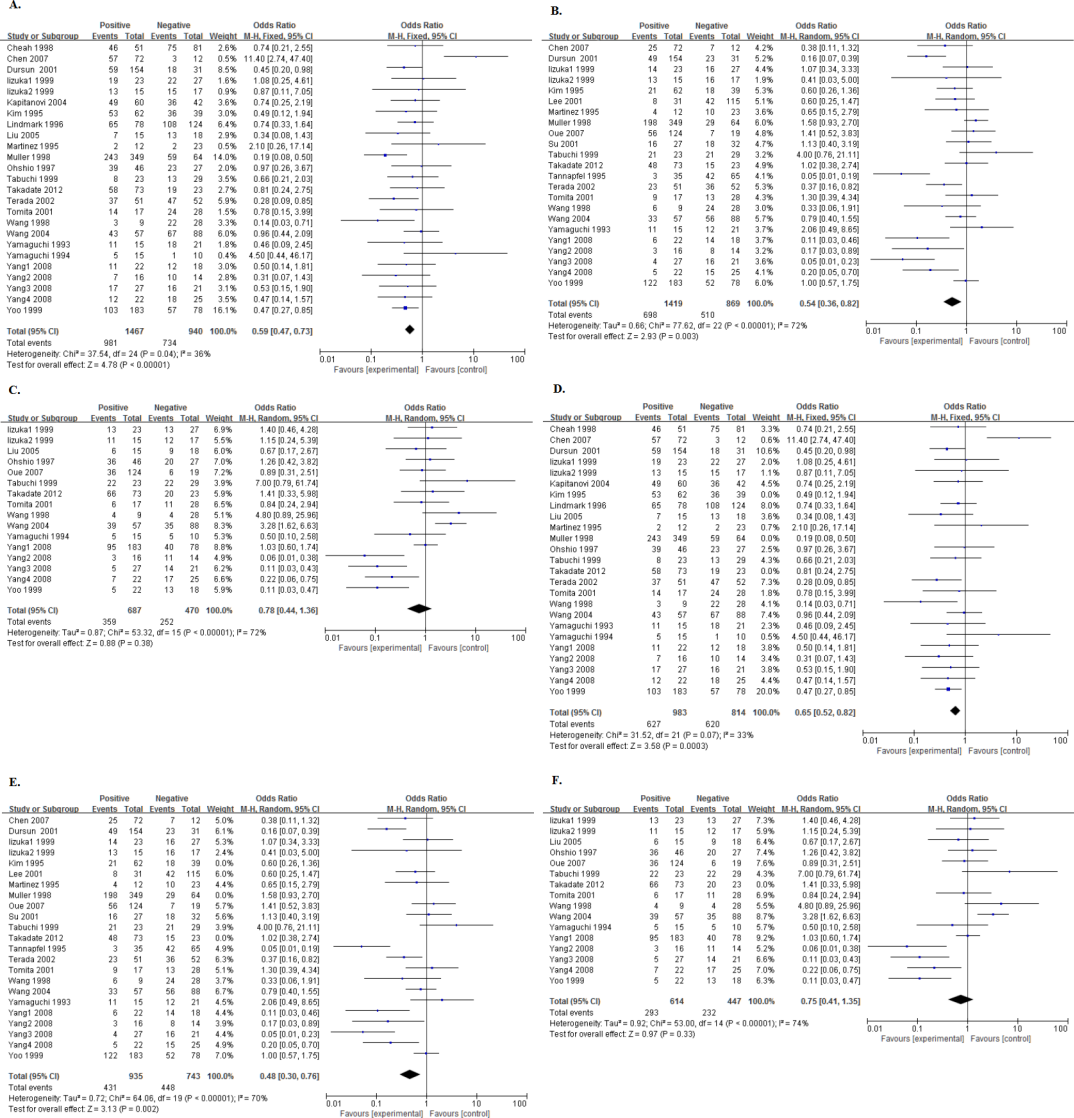
Forest plot of odds ratio (OR) for the association between NME1 overexpression and clinical pathological factors in patients with random effects model. A. The ORs for tumor differentiation; B. The ORs for N status; C. The ORs for TNM stage; D. The ORs for tumor differentiation without low cut-offs; E. The ORs for N status without low cut-offs; F. The ORs for TNM stage without low cut-offs.

One article[48] investigated four types of digestive system cancers, so we marked them as Yang1, Yang2, Yang3 and Yang4. With a low heterogeneity (I^2^ = 36%, Ph = 0.04), the pooled OR being 0.59 (95%CI:0.47–0.73, P<0.00001. Fig 6A.) of 25 cohorts showed that high expression of NME1 was significantly associated with well tumor differentiation. Though with heterogeneity (I^2^ = 72%, Ph<0.00001), 23 cohorts presented data about NME1 expression and N status, and a combined OR being 0.54 (95%CI:0.36–0.82, P = 0.003. Fig 6B) indicated that the positive relationship between increased NME1 expression and negative N status. A pooling OR without any significance, was produced by 16 cohorts which reported the association between NME1 expression and TNM stage (OR = 0.78, 95%CI:0.44–1.36, P = 0.38. Fig 6C).

Then, we delete the studies[33,34,47] which had low cut-offs, to obtain more precise pooled estimates. No significant differences could be found in these new ORs (Fig 6).

### Relationship of NME1 expression with clinical pathological factors by tumor type

**Fig 7:**
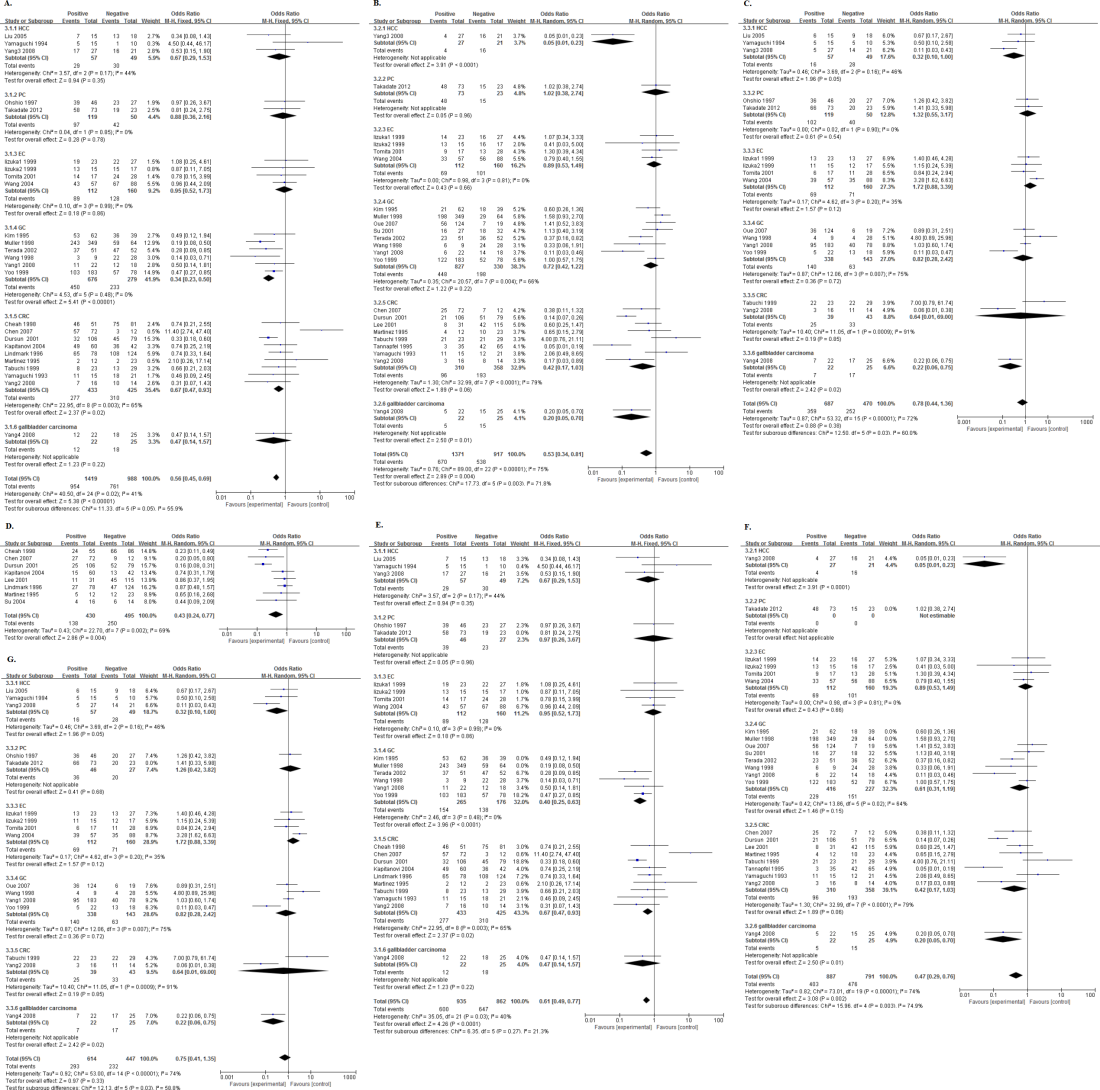
Forest plot of odds ratio (OR) for the association between NME1 overexpression and clinical pathological factors in patients with different tumor types with random effects model. A. The ORs for tumor differentiation; B. The ORs for N status; C. The ORs for TNM stage; D. The ORs for Dukes’ stage; E. The ORs for tumor differentiation without low cut-offs; F. The ORs for N status without low cut-offs; G. The ORs for TNM stage without low cut-offs.

There were 3, 2, 4, 6, 9 and 1 cohorts reporting the data of NME1 expression and tumor differentiation of the patients with HCC, PC, EC, GC, CRC and GBC, respectively. Only in GC and CRC, increased NME1 expression was significantly associated with well tumor differentiation (OR = 0.34, 95%CI:0.23–0.50, P<0.00001, I^2^ = 0%, Ph = 0.48 and OR = 0.67, 95%CI:0.47–0.93, P = 0.02, I^2^ = 65%, Ph = 0.003, respectively. Fig 7A). However, the relationship between NME1 expression and N status failed to obtain the statistical significance in any tumor type (Fig 7B). It was the same to the association between NME1 expression and TNM stage (Fig 7C). In the colorectal cancer, there were eight cohorts reporting the relationship between the expression of NME1 and Dukes’ stage. Though with heterogeneity (I^2^ = 69%, Ph = 0.002), the combined OR being 0.43 (95%CI:0.24–0.77, P = 0.004. Fig 7D), indicated that elevated NME1 expression was significantly related to Dukes’ stage A and B.

Then, we deleted the three cohorts[33,34,47] again, and failed to find any significant difference in all of these three factors as well (Fig 7).

### Subgroup analyses

Because of too few articles or no heterogeneity, we only conducted stratifying analysis for gastric cancer and colorectal cancer in OS, N status and Dukes’ stage. Main results of subgroup analysis were listed in Tables 3, 4 and 5. Except the “> 2000” of gastric cancer with a significant estimate (OR = 0.39, 95%CI:0.19–0.80, P = 0.01), none of the other subgroups had statistical significance (Table 3). And with both of the “≦2000” and “> 2000” having a I^2^<50%, the “years” might be the source of heterogeneity of overall survival in gastric cancer. Although we obtained quite a few highly significant estimates in the following subgroups (Tables 4 and 5), we couldn’t find any possible source of heterogeneity in N status and Dukes’ stage.

**Table 3:** Meta-analysis estimates for overall survival

		Gastric cancer					Colorectal cancer			
Factor	No. of studies	OR(95%CI)	P	I^2^(%)	Ph	No. of studies	OR(95%CI)	P	I^2^(%)	Ph
All studies	5	0.75 [0.34, 1.62]	0.46	79	0.0006	7	0.57 [0.31, 1.04]	0.07	72	0.002
Study region										
Asian	4	0.55 [0.23, 1.28]	0.17	72	0.01	3	0.87 [0.53, 1.45]	0.60	0	0.51
Caucasian	1	2.08 [1.21, 3.58]	0.008	-	-	4	0.39 [0.14, 1.11]	0.08	83	0.0005
Sample size											
<100	1	0.15 [0.04, 0.56]	0.004	-	-	3	0.50 [0.15, 1.59]	0.24	75	0.02
≥100	4	1.03 [0.53, 1.99]	0.93	71	0.02	4	0.61 [0.28, 1.36]	0.23	76	0.005
Years										
≦2000	2	1.62 [0.99, 2.65]	0.06	40	0.20	4	0.56 [0.26, 1.21]	0.14	68	0.03
> 2000	3	0.39 [0.19, 0.80]	0.01	29	0.25	3	0.57 [0.17, 1.91]	0.36	83	0.003

**Table 4:** Meta-analysis estimates for N status

		Gastric cancer					Colorectal cancer			
Factor	No. of studies	OR(95%CI)	P	I^2^(%)	Ph	No. of studies	OR(95%CI)	P	I^2^(%)	Ph
All studies	8	0.72 [0.42, 1.22]	0.22	66	0.004	8	0.42 [0.17, 1.03]	0.06	79	<0.0001
Study region										
Asian	7	0.62 [0.35, 1.07]	0.09	57	0.03	5	0.77 [0.30, 1.99]	0.59	61	0.04
Caucasian	1	1.58 [0.93, 2.70]	0.09	-	-	3	0.16 [0.05, 0.50]	0.002	70	0.04
Sample size										
<100	3	0.37 [0.08, 1.64]	0.19	71	0.03	4	0.99 [0.27, 3.59]	0.98	64	0.04
≥100	5	0.89 [0.53, 1.50]	0.66	63	0.03	4	0.21 [0.08, 0.56]	0.002	76	0.006
Years										
≦2000	4	0.96 [0.57, 1.61]	0.87	48	0.12	4	0.70 [0.10, 5.00]	0.72	86	<0.0001
> 2000	4	0.54 [0.20, 1.47]	0.23	73	0.01	4	0.27 [0.12, 0.62]	0.002	61	0.05

**Table 5:** Meta-analysis estimates for Dukes’ stage

		Colorectal cancer			
Factor	No. of studies	OR(95%CI)	P	I^2^(%)	Ph
All studies	8	0.43 [0.24, 0.77]	0.004	69	0.002
Study region					
Asian	4	0.38 [0.18, 0.81]	0.01	52	0.10
Caucasian	4	0.49 [0.19, 1.26]	0.14	81	0.001
Sample size					
<100	3	0.66 [0.34, 1.28]	0.22	0	0.85
≥100	5	0.37 [0.17, 0.80]	0.01	81	0.0004
Years					
≦2000	3	0.50 [0.20, 1.29]	0.15	73	0.02
> 2000	5	0.39 [0.18, 0.88]	0.02	71	0.009

### Publication bias

**Fig 8:**
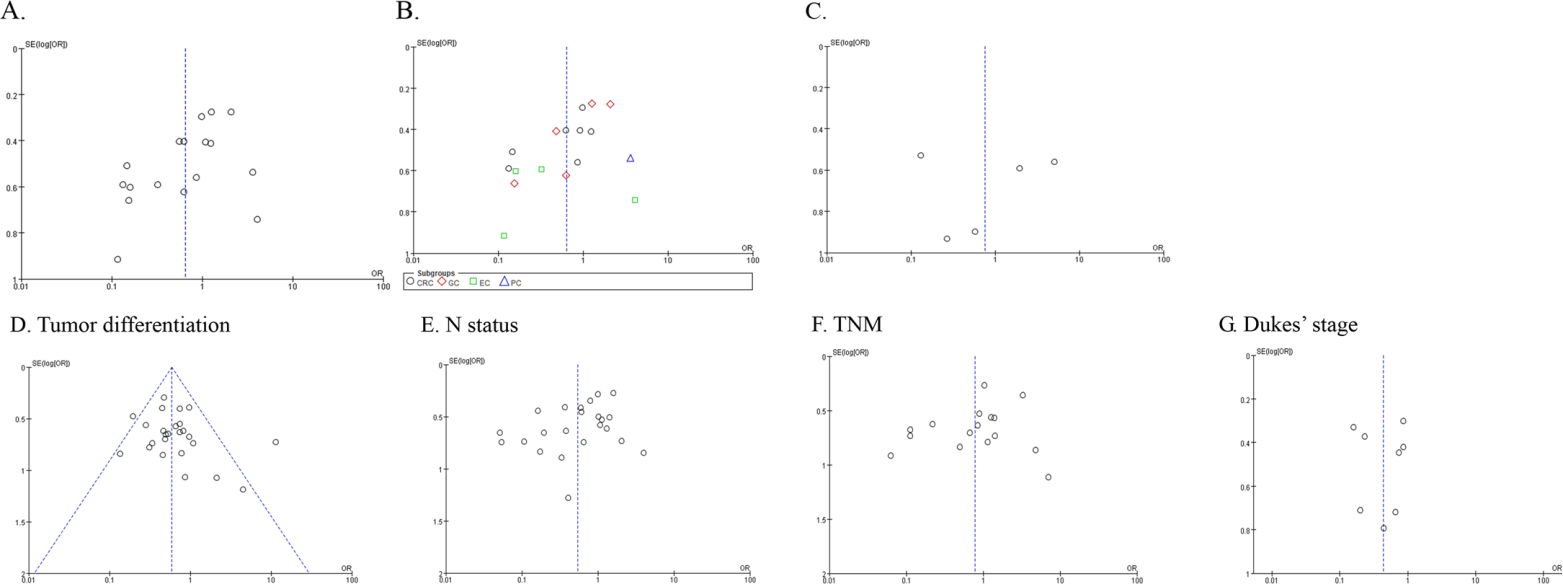
Funnel plot for NME1 expression. A. and B. OS; C. DFS; D. tumor differentiation; E. N status; F. TNM stage; G. Dukes’ stage.

A funnel plot was used to discover the possibility of publication bias. And no obvious asymmetry was observed in funnel plots (Fig 8). Except the P value for NME1 and OS, the P value of Egger’s test for others also indicated no obvious publication bias (P>0.05. S2 Table). Then, we carried out the Egger’s test for NME1 and OS by tumor type. All of the P value for CRC, GC and EC indicated that there was no obvious publication bias (P = 0.116, 0.061 and 0.871, respectively. S2 Table).

### Sensitivity analysis

**Fig 9:**
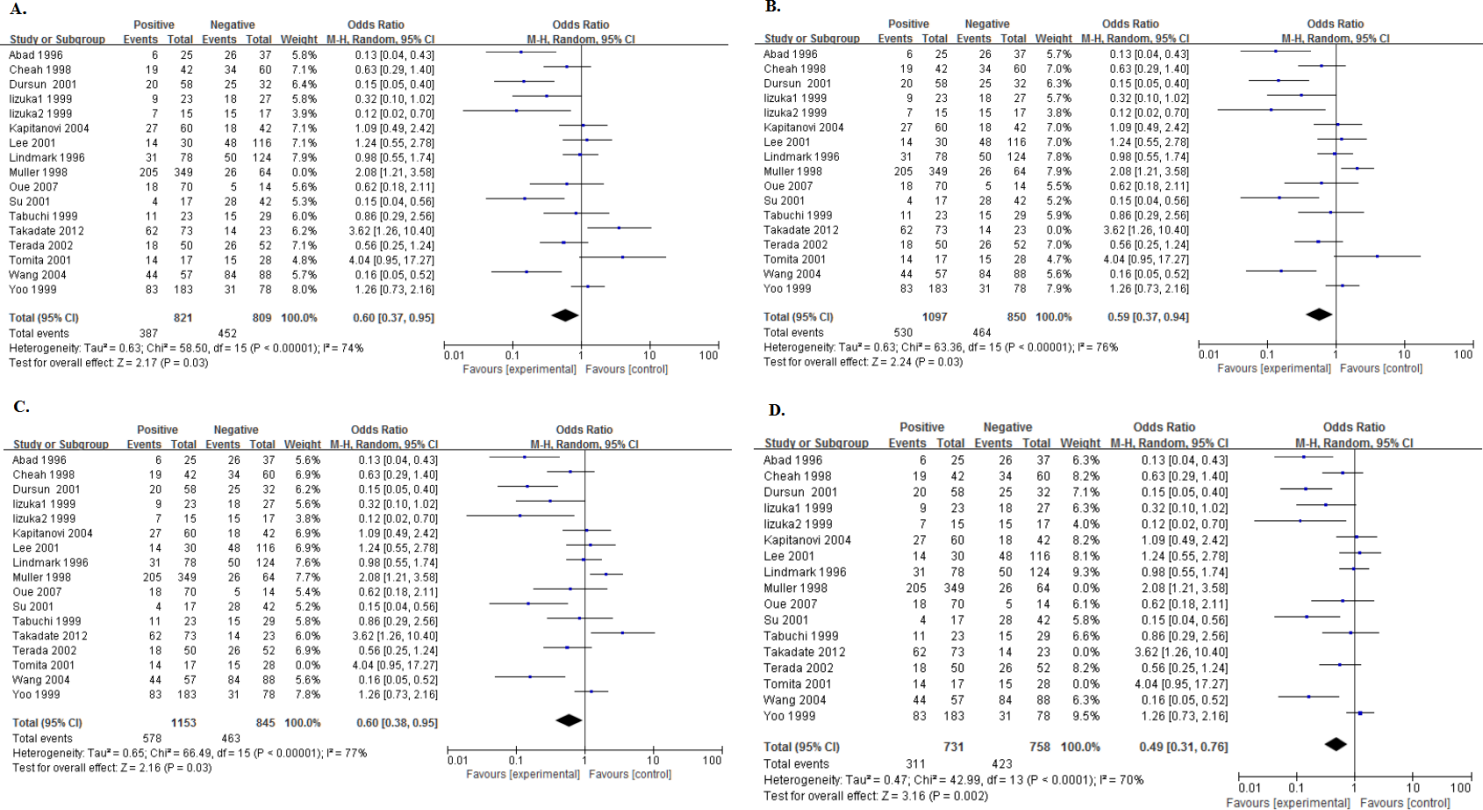
Forest plot of odds ratio (OR) for sensitivity analysis of the association between NME1 overexpression and overall survival (OS) in patients with random effects model. A. The ORs for OS without “Muller 1998”; B. The ORs for OS without “Takadate 2012”; C. The ORs for OS without “Tomita 2001”; D. The ORs for OS without these three studies.

To test the stabilization of our results, we deleted one individual cohort each time and calculated the pooled ORs of the studies left. No significant differences were observed between the corresponding results and the overall results (data not shown), except the three new combined ORs of overall survival (Fig 9). Among these three studies[34,40,47], two[34,47] had a low cut-off as written before, and both obtained a new OR with significance when removing them individually (OR = 0.60, 95%CI:0.37–0.95, P = 0.03. Fig 9A; OR = 0.59, 95%CI:0.37–0.94. Fig 9B). When excluding the left one[40], which had a moderate cut-off, we also gained a significant OR being 0.60 (95%CI:0.38–0.95, P = 0.03. Fig 9C). After removing these three studies[34,40,47] and recalculating the new pooled OR, a significant estimate was produced (OR = 0.49, 95%CI:0.31–0.76, P = 0.002. Fig 9D), but still had a high heterogeneity (I^2^ = 70%, Ph<0.0001).

## Discussion

Meta-analysis of biomarker prognostic value was attached to molecular pathological epidemiology (MPE), an integrative transdisciplinary science which was commonly applied to research on various carcinomas and mainly based on the unique disease principle and continuum theory[49]. Thus, to explore potential tumor biomarkers, we combined 28 articles with 2904 patients, and conducted this meta-analysis. Despite the total pooled ORs of OS and DFS had no significance in statistics, after removing the three studies, whose cut-offs were too low as compared with others[34,47], or with a low NOS score[24], the new pooled estimates revealed that elevated NME1 expression predicted better OS and DFS (OR = 0.59, 95%CI:0.38–0.92, P = 0.02; OR = 0.20, 95%CI:0.09–0.45, P<0.0001, respectively). However, when we stratified the pooled data by tumor types, only one OR combined by two studies[25,27], had a P<0.05 (OR = 0.23, 95%CI:0.06–0.94, P = 0.04). Thus, it was difficult for us to identify whether high expression of NME1 is associated with better prognosis.

Among the clinical pathological factors evaluated, we could find that elevated NME1 expression was related to well differentiation and N status, but not to TNM stage, in patients with digestive system cancers. Then, we stratified the pooling data by tumor types again and we discovered statistical significance only in GC and CRC. The relationship between enhanced expression of NME1 and negative N status is false in all types of digestive system cancers. In addition, we revealed that elevated NME1 expression was significantly related to Dukes’ stage A and B. Hence, the association between NME1 overexpression and better clinicopathological outcome could be proven partly, through this meta-analysis.

In our subgroup analysis, we only analyzed the OS and N status in gastric cancer and colorectal cancer, and Dukes’ stage in colorectal cancer. And we only found that the subgroup “years” might be the source of heterogeneity of overall survival in gastric cancer, in view of the two I^2^ in this subgroup both lower than 50%. At the same time, in this subgroup, we discovered that the “> 2000” had a significance in statistics, while the “≦2000” gained a P = 0.06. This could also be found in N status and Dukes’ stage in colorectal cancer (in the “> 2000”, OR = 0.27, 95%CI:0.12–0.62, P = 0.002, and OR = 0.39, 95%CI:0.18–0.88, P = 0.02, respectively; and in the “≦2000”, OR = 0.70, 95%CI:0.10–5.00, P = 0.72, and OR = 0.50, 95%CI:0.20–1.29, P = 0.15, respectively). Maybe, with the development of science and technology, the results would be more and more precise. Likewise, this revealed that high NME1 expression might be associated with better overall survival, negative lymph node metastasis, and Dukes’ stage A and B.

Loss of heterozygosity (LOH) and Microsatellite instability (MSI) of NME1 were two independent genetic pathways and crucial mechanisms in the development and progression of digestive system cancers[50–53]. LOH mostly arose in the late period of sporadic colon cancer and endowed it with high aggressive and poor prognosis, while NME1 overexpression suppressed colon cancer metastasis and promoted prognosis of sporadic colon cancer patients, effectively[52]. In gallbladder carcinoma, MSI was an early stage molecule marker and LOH was a molecule marker for the deteriorism which could inhibit the expression of NME1 in local tissues[51]. Also, the frequency of NME1 protein in stages I + II was higher than that in stages III + IV; that in well differentiation cases was higher than in poor differentiation cases; and that in the group of metastasis was higher than that with metastasis significantly[51,52]. These findings revealed that LOH and MSI of NME1 were both associated with worse prognosis and clinical pathological factors. In other cancers, regulating the Ras-MAPK pathway is another key molecular function of NME1[54,55]. Upregulation of NME1 inhibited KSHV-induced Ras-BRaf-MAPK pathway activation, and overexpression of NME1 by 5-aza-2’-deoxycytidine reduced KSHV-induced cell invasiveness[54]. Thus, transferring and overexpressing NME1 into animals, maybe suppressed the growth and development of tumors and obtained a better prognosis. Li[56] used an adeno-associated virus (AAV) to transfer NME1 gene into the mice, and led to the 60% reduction in the number of animals developing liver metastasis. In addition, a significant NME1-induced enrichment for members of the CDC42 signaling cascade was identified, using Fisher’s exact test (p<0.014),including ARPC5L, CDC42, CDC42EP2, FNBP1L, HLA-DOA, HLA-F, HLA-G, ITGB1, JUN, MYL7, MYL10, MYL12A and RASA1, all of which were regulated by NME1, and linked to metastasis and outcome of patients with melanoma and breast carcinoma[10]. However, few clinically relevant therapeutic targets had been developed from these known substrates of NME1[55].

Admittedly, our meta-analysis is subject to a few limitations. Firstly, because of several antibodies recognising both NME1 and NME2, we didn’t use the articles which only reported NM23, but not NME1. These articles couldn’t explain the effect of overexpression of NME1, but excluding them also could cause selection bias or else; Secondly, all of the enrolled studies were retrospective, and some biases, such as selection bias, misclassification bias and information bias, might be present in the meta-analysis; Thirdly, the ORs of OS or DFS, were all estimated from the Kaplan-Meier curves in this meta-analysis. This estimate could produce biases inevitably. Because no studies on NME1 used HRs to evaluate OS or DFS, and the estimated HRs calculated through K-M curves were inaccurate, we used ORs to assess OS or DFS; Fourthly, all cohorts we included, was investigated by IHC. Maybe other methods could also indicated the prognostic value of NME1 expression. In addition, though with a total of 28 cohorts, which reported patients with digestive system cancers, certain tumor types, like pancreatic cancer and esophagus cancer, had too few cohorts; and despite no publication bias was detected in funnel plots, evidence of publication bias in our formal statistical test was almost always underpowered with only 28 studies. Thus, further studies were required to be carried out in the future. Besides, we only adopted articles written in English. This could lose some available studies in other languages. And some unpublished studies could also be ignored. The last but not least, in this meta-analysis, our results, especially in overall survival, failed to reveal its good prognostic value in patients with digestive system cancers. Fortunately, we discovered that elevated NME1 expression might be related to well tumor differentiation and N status. Hence, we will continue searching articles in the following years and make updates immediately. In a word, our results might be flawed, to some extent.

## Conclusions

In this systematic review with meta-analysis, although we failed to identify whether the elevated NME1 expression was associated with a poor or well prognosis in patients with digestive system neoplasms, our results indicated that NME1 expression might be related with the clinicopathologic factors of digestive system cancers, including tumor differentiation, N status, and Dukes’ stage. Thus, further studies should be performed to confirm our conclusion and explore its molecular functions.

## Supporting Information

S1 Table PRISMA Checklist. (DOC)

S2 Table Egger’s test. (DOC)

S3 Table Clinical Studies Checklist. (DOCX)
