# Supplementary figures and images for: Prognostic Value of NME1 (NM23-H1) in Patients with Digestive System Neoplasms: A Systematic Review and Meta-Analysis
Source: PLoS One. 2016 Aug 12;11(8):e0160547. doi: 10.1371/journal.pone.0160547 (PMC4982620; doi:10.1371/journal.pone.0160547)

1. OS

All


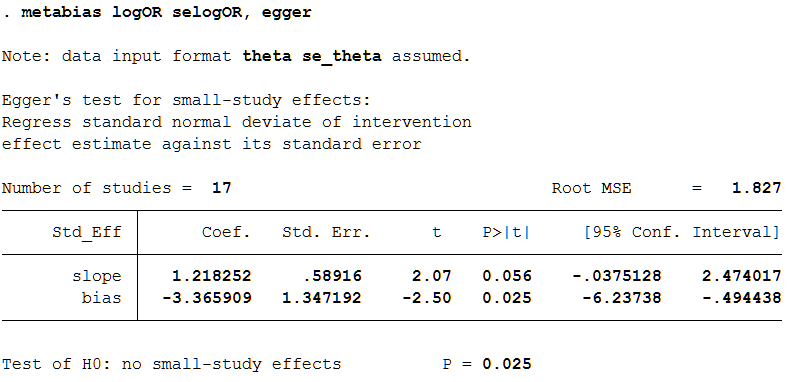


CRC


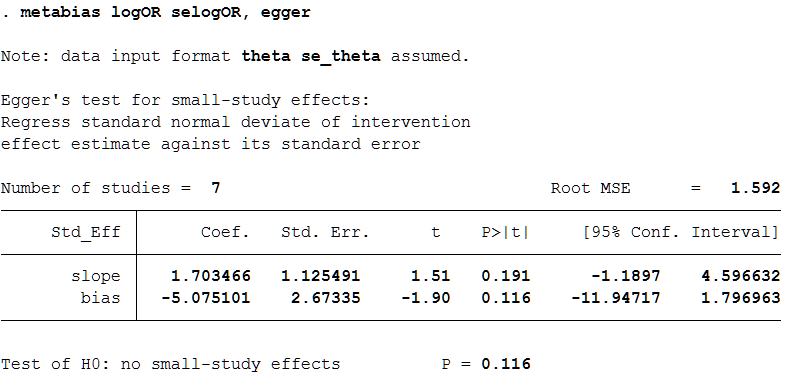


GC


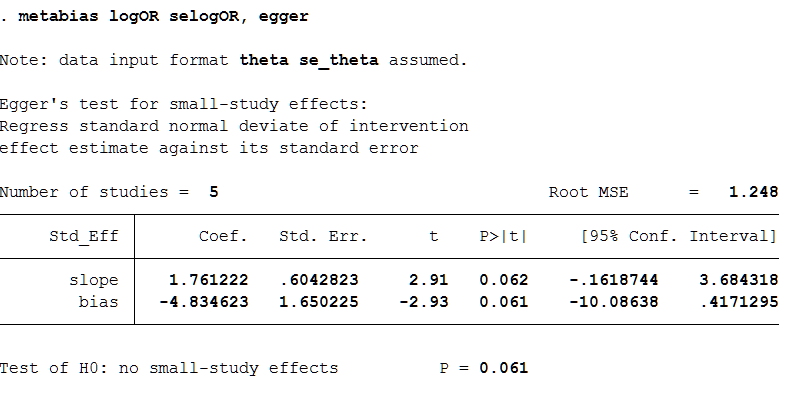


EC


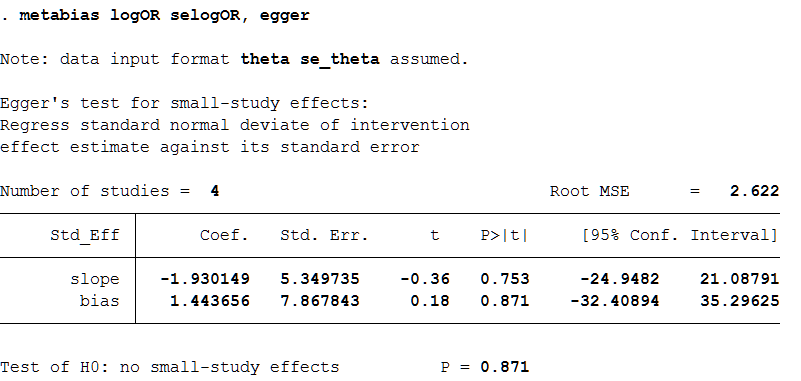


1. DFS


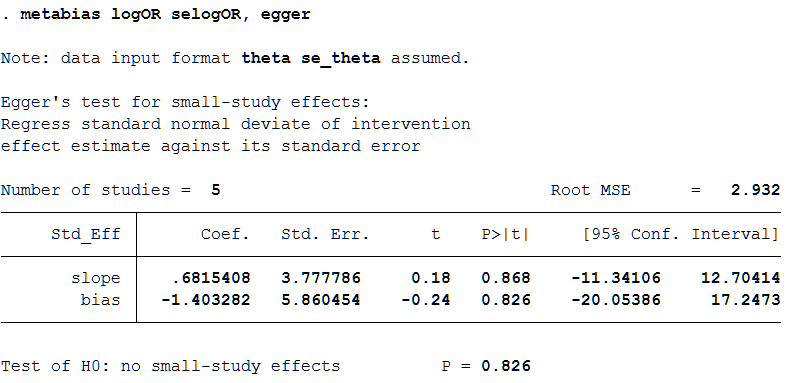


1. Differentiation


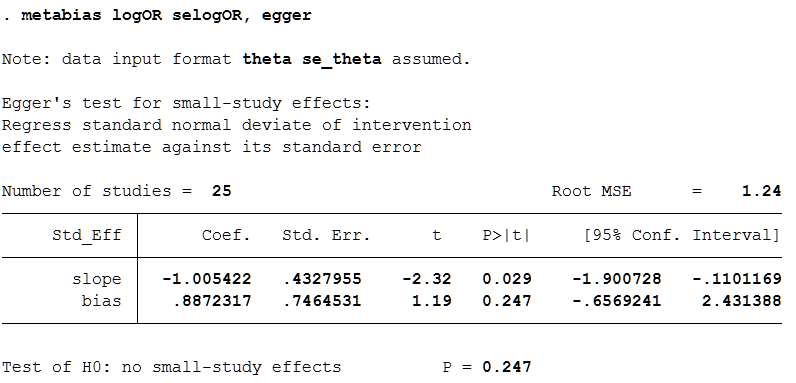


1. N


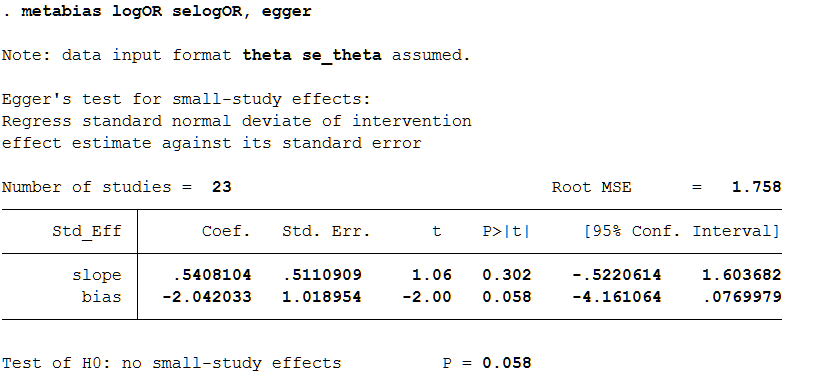


1. TNM


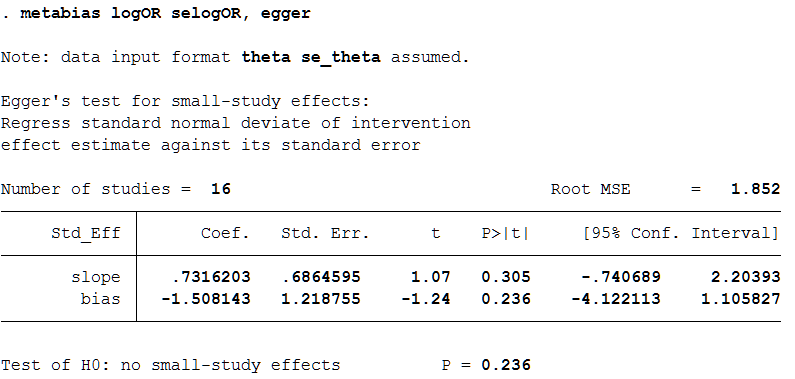


1. Dukes’ stage


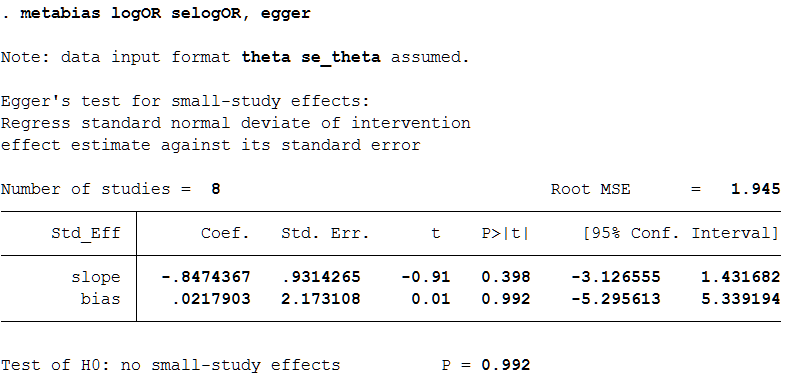

Supplement: S2 Table — (DOC) [file pone.0160547.s002.doc]
